# Overnight polysomnography and the recording of sleep and sleep-related respiration in orchestra musicians – possible protective effects of wind instruments on respiration

**DOI:** 10.1371/journal.pone.0231549

**Published:** 2020-04-15

**Authors:** Naima Laharnar, Stefanie Uibel, Corin Hild, Martin Glos, Thomas Penzel, Ingo Fietze

**Affiliations:** 1 Interdisciplinary Sleep Center, Charité–Universitätsmedizin Berlin, Berlin, Germany; 2 Medical Clinic III / Cardiology, University Hospital Frankfurt, Frankfurt am Main, Germany; 3 Saratov State University, Saratov, Russia; Harper University Hospital, UNITED STATES

## Abstract

Our study is the first to objectively assess sleep and sleep-related respiration in orchestra musicians. We hypothesized low sleep quality due to high work demands and irregular work-sleep schedules, and a better respiration for wind instrument (WI) players than string instrument (SI) players due to habitual upper airway muscles training. We recorded overnight polysomnography with 29 professional orchestra musicians (21 men, 14 WI/ 15 SI). The musicians presented a sleep efficiency of 88% (IQR 82–92%) with WI having a significant higher sleep efficiency than SI (89%, 85–93% vs. 85%, 74–89%; *p = 0*.*029*). The group had a total sleep time around 6 hours (377min, 340-421min) with signs of increased NREM 1 (light sleep) and decreased REM (dream sleep). The musicians displayed an apnea-hypopnea-index of 2.1events/hour (0.7–5.5) and an oxygen saturation of 98% (97–100%). While SI player exhibited declining sleep-related respiration with age (breathing events: r = 0.774, *p = 0*.*001*, oxygen: r = -0.647, *p = 0*.*009)*, WI player showed improved respiration with age (breathing events: r = -0.548, *p = 0*.*043*; oxygen: r = 0.610, *p = 0*.*020)*. Our study is the first objective investigation of sleep pattern and respiration during sleep with overnight polysomnography in professional orchestra musicians. While sleep and respiration were unexpectedly good, our results revealed possible signs of sleep deprivation and an interesting age-related pattern on respiration depending on instrument. While sample size was small and results modest, these findings present first objective evidence towards the assumption that habitual playing of a WI–and training of the upper airway muscles–may have a protective effect on respiration.

## Introduction

While sleep has a restorative function, [1] sleep deprivation can lead to daytime sleepiness, mood changes, cognitive deficits, impaired work ability, accidents, immune deficiency, and cardiovascular risk [2–5]. Sleep data of healthy sleepers is lacking,^3^ but studies show that healthy sleepers exhibit a sleep efficiency (SE, ratio of time asleep to total time in bed) above 85% with a seven to eight hours sleep duration [6–8]. Professions with irregular schedules and high stress (e.g., shift workers, professional athletes) may experience reduced sleep duration and quality, resulting in an impaired working ability [9,10]. Orchestra musicians undergo similar extraordinary demands with extended working hours and performances in the late evening. With expectations of precise physical and psychological performance, they are compared to top-ranking athletes [11,12]. A Norwegian survey revealed that professional musicians were dissatisfied with their sleep and suffered from increased insomnia [13]. However, objective sleep patterns of professional musicians have not yet been investigated.

While working conditions of professional musicians may negatively affect their sleep patterns, recent studies discovered that playing instruments that require use of certain muscles may have a positive effect on sleep-disordered breathing events [14–16]. Obstructive sleep apnea (OSA) is a chronic sleep-disordered breathing condition caused by recurrent upper-airway collapse during sleep [17,18]. With a high prevalence of 46% to 75%, OSA is associated with daytime sleepiness, car accidents, metabolic and cardiovascular diseases [19,20]. Even people without OSA experience increased breathing events during their REM (rapid eye movement) sleep phase, thus decreasing their sleep quality [21]. The habitual use of a didgeridoo, a wind instrument (WI, sounded by the player’s breath) of the indigenous Australians, has been shown to reduce sleep-disordered breathing events [14,15]. Training the airway muscles that control airway dilatation and wall stiffening may diminish collapse of oropharyngeal muscles at night and improve sleep [22–24]. Only two web-based surveys investigated such a preventive effect in professional musicians with rather inconclusive results [16,25,26].

Our study is the first to objectively record overnight sleep and sleep-related respiration in orchestra musicians. We hypothesized that sleep quality is low due to sleep deprivation and irregular sleep-wake schedule, and that WI players exhibit better respiration than SI (string instrument) players due to habitual training of the upper airway muscles.

## Methods

### Recruitment

We recruited members of the orchestra of the Berlin State Opera in Germany (Deutsche Staatsoper Berlin, 112 members) during performance breaks and via posted advertisements. Participants needed to be active full-time musicians between 18 and 65 years. Exclusion criteria included sleep-affecting medical conditions and medications, sleep-wake disorders, respiratory pathology, psychiatric-neurological, psychological, or internal diseases affecting compliance, and involvement in another study. Recruitment was conducted for three years. All participants signed informed consent. The study protocol was approved by the Institutional Review Board at the Charité–Universitätsmedizin Berlin (EA1/066/099).

### Procedure and data

Overnight polysomnography (PSG, SOMNOcheck 2 R&K, Weinmann, Hamburg, Germany) was conducted at the participant’s home for one night. Sensors were attached in the evening by a trained sleep specialist and removed in the morning by the participant. The device recorded two electroencephalogram derivations, two chin and four tibialis electromyogram derivations, two electrooculogram derivations, air flow, thoracic and abdominal movements, three electrocardiogram derivations, and oximetry data. In the morning, participants completed four sleep questionnaires: The Pittsburgh Sleep Quality Index (PSQI) measured sleep quality with scores above 5 indicating poor sleep quality [27]. The Epworth Sleepiness Scale (ESS) measured excessive daytime sleepiness with scores above 10 indicating excessive daytime sleepiness [28]. The Morningness-Eveningness Questionnaire (D-MEQ) determined the circadian rhythm [29]. Scores between 16 and 41 indicated evening type, scores between 42 and 58 intermediate type, and scores 59 to 86 morning type. The FEPS questionnaire ("Fragebogen zur Erfassung allgemeiner und spezifischer Persönlichkeitsmerkmale Schlafgestörter") is gender specific and divided into two subscales [30]. "Focusing" referred to sleep-related cognitive arousals due to increased attention towards sleep leading to difficulties initiating or maintaining sleep, scores 28 to 36 (women) and 21 to 36 (men) were unhealthy. "Brooding" referred to general cognitive arousals due to uncontrolled repetitively thinking about unresolved problems, scores 38 to 49 (women) and 31 to 45 (men) were unhealthy. Combination of high focusing and brooding scores indicated a primary insomnia.

### Sleep data and data analysis

Visual scoring of sleep and respiration were performed by certified sleep technologists according to AASM 2007 (American Academy of Sleep Medicine) criteria [31]. The apnea-hypopnea index (AHI, events per hour) was calculated with an AHI ≥ 5 indicating a form of OSA. Data analysis was performed with SPSS (IBM SPSS Statistics, version 23). Due to the small sample size, a non-parametric analysis and a rather descriptive presentation of the results were chosen. Subgroups were formed to investigate effects of instrument (WI vs. SI) and age (<41 years vs. ≥41 years). Data were presented as medians (IQR, interquartile range) or numbers (%). Mann-Whitney U tests, Chi-square, and Spearman correlation were conducted, *p*-values <0.05 were considered statistically significant. Additionally, the Quade’s Test, a non-parametric rank analysis of covariance, was conducted to investigate differences between instrument groups while controlling for age [32]. Here, the dependent variables (objective sleep, subjective sleep, respiration) and covariate (age) were ranked, the residuals of a linear regression with ranked dependent variables and ranked covariate were saved, and an ANOVA with the residuals and the grouping variable (instrument group) was run. The F-test resulting from the ANOVA is the F statistic used by Quade.

## Results

### Sample

Thirty-four participants were recruited, five were excluded (battery failure, loosened electrodes, sleep time less than ten minutes). The final 29 participants (21 men, Table 1) had a median age of 41 years (IQR: 33–50, range: 26–56 years) and a median body-mass-index (BMI) of 25 kg/m^2^ (IQR: 22–26, range: 19–34 kg/m^2^). Ten participants were overweight (BMI ≥ 25 kg/m^2^) with two being obese (BMI ≥ 30 kg/m^2^). Participants practiced a median of 2.5 hours per day (IQR: 2.0–3.0, range: 1.0–5.0 hours). They had played the instrument for a median of 27 years (IQR: 22–35, range: 15–48 years) and had started playing the instrument at a median age of 11 years (IQR: 6.5–16.5, range: 5–19 years). There was a significant high positive correlation between age and years of practice (r = 0.90; *p = 0*.*000*). The sample included 14 WI players (3 horns, 1 trumpet, 1 trombone, 3 flutes, 2 clarinets, 3 oboes, 1 bassoon) and 15 SI players (2 bass, 7 violins, 3 violas, 3 cellos). There were no significant differences between the instrument groups except for age of first practice. SI players started at age 7.5 years significantly earlier than WI players at age 15.0 years.

**Table 1:** Participant characteristics, presented are n (%) or median (IQR).

	ALL n = 29	WI n = 14 (48%)	SI n = 15 (52%)	*p*
**Men**	21 (72%)	11(79%)	10 (67%)	0.682
**Age (years)**	41.0 (32.5–49.5)	45.0 (33.8–50.3)	40.0 (29.0–47.0)	0.234
**Age <41 years**	14 (48%)	5 (36%)	9 (60%)	0.272
**Age ≥41 years**	15 (52%)	9 (64%)	6 (40%)	
**BMI *(*kg/m**^**2**^***)***	24.6 (21.7–26.3)	24.7 (22.1–26.7)	24.2 (20.8–25.9)	0.538
**BMI ≥25**	10 (40%)	6 (46%)	4 (33%)	0.905
**BMI ≥30**	2 (4%)	1 (8%)	1 (8%)	*n/a*
**Practice (h/day)**	2.5 (2.0–3.0)	2.5 (2.0–2.5)	3.5 (1.5–4.8)	0.177
**Practice (years)**	27.0 (22.0–35.0)	25.0 (23.0–32.0)	27.0 (19.8–42.0)	0.809
**Age of first practice (years)**	11.0 (6.5–16.5)	15.0 (10.0–18.0)	7.5 (6.0–12.0)	**0.029**
**Allergies**^a^ **(yes)**	13 (45%)	4 (29%)	9 (60%)	0.057
**Alcohol**^b^ **(yes)**	7 (24%)	2 (14%)	5 (33%)	0.183
**Medication**^c^ **(yes)**	7 (24%)	5 (36%)	4 (27%)	0.652
**Previous Upper Airway Ops (yes)**	9 (31%)	5 (36%)	4 (27%)	0.700
**Sports (yes)**	14 (48%)	8 (57%)	6 (40%)	0.640

WI, wind instrument; SI, string instrument; BMI, body mass index in kg/m^2^; n/a, not applicable. *p*-values represent chi-square tests for dichotomous variables and Mann-Whitney U test for continuous variables, values above 0.05 are considered significant.

^a^Allergies include hayfever, cat hair, mold, ciprobay, penicillin, paracetamol, tropical fruits, pollen, birch, grass.

^b^Alcohol consumption at day of examination.

^c^Medication include thyroxin, birth control, nasal spray, nexium (stomach acid reducer), delix (hypertension), ezetrol (high cholestoral), salbutamol (asthma).

### Objective sleep quality

The group (Table 2) had a median SE of 88% (IQR: 82–92%, range: 61–96) with a median TST (total sleep time) of 377 min (IQR: 340–421, range: 317–492 min). WI players had a significant higher SE than SI players (*p = 0*.*029*). Controlling for age, the Quade’s Test confirmed the significant difference in SE between instrument groups (F = 5.955, *p = 0*.*022*). The difference was strongest for the younger age group below 41 years (WI, n = 5: SE: 92%, IQR: 89–94% vs. SI, n = 9: 85%, 76–89%, *p = 0*.*029*). Independent of instrument, the older age group had significant lower REM-L than the younger age group (<41 years, n = 15: 86.3min, 70.5–116.5min vs. ≥41 years, n = 14: 66.5min, 61.0–73.5min; *p = 0*.*041*). Sleep stages did not differ significantly between instruments. Participants spent a median of 7% (IQR: 5–12) in NREM 1 (light sleep 1), 49% (43–55) in NREM 2 (light sleep 2), 20% (13–26) in SWS stage (short wave sleep, deep sleep), and 16% (12–19) in REM stage (dream sleep).

**Table 2:** PSG sleep data, presented are median and interquartile range.

	ALL n = 29	WI n = 14 (48%)	SI n = 15 (52%)	*p*
**SE (%)**	87.9 (82.1–91.6)	89.2 (85.6–93.3)	85.1 (73.6–89.3)	**0.029**
**TIB (min)**	426.0 (397.5–463.3)	425.0 (390.0–461.5)	427.0 (402.0–501.0)	0.733
**TST (min)**	377.0 (340.0–421.0)	379.0 (345.5–421.5)	377.0 (337.0–423.0)	0.813
**SL2 (min)**	24.5 (7.3–40.3)	20.5 (6.1–32.9)	24.5 (12.0–65.0)	0.290
**REM-L (min)**	71.0 (61.8–97.3)	70.8 (61.8–111.8)	74.0 (61.5–91.5)	0.983
**WASO (min)**	26.5 (14.3–41.5)	20.8 (11.3–29.4)	31.5 (16.0–46.0)	0.123
**NREM 1 (% of SPT)**	7.1 (4.8–11.8)	7.0 (4.5–12.6)	7.5 (4.7–12.1)	0.983
**NREM 2 (% of SPT)**	48.9 (43.2–55.4)	51.2 (44.9–60.6)	47.2 (41.1–51.9)	0.085
**SWS (% of SPT)**	20.3 (12.6–26.0)	16.7 (10.5–26.1)	21.5 (15.9–26.1)	0.331
**REM (% of SPT)**	15.9 (12.0–19.4)	16.3 (11.9–19.4)	15.8 (12.1–18.8)	0.847

SE, sleep efficiency, percentage of total sleep time to total time in bed; TIB, time in bed from lights off to lights on; TST, total sleep time through last epoch of sleep without waking time; SL2, sleep onset latency to sleep stage 2, time from light off till first sleep epoch of at least 30 seconds of the second sleep stage; REM-L, REM sleep onset latency, time from sleep onset till first epoch of rapid eye movement sleep stage epoch; WASO, wake after sleep onset; SPT, sleep period time, total sleep time including wake time after onset; NREM 1, non-rapid eye movement sleep, sleep stage 1; NREM 2, non-rapid eye movement sleep, sleep stage 2; SWS, slow wave sleep, sleep stages 3 and 4; REM, rapid eye movement, sleep stage 5; wind, wind instrument players, string, string instrument players. *p*-values represent Mann-Whitney U test. Highlighted are significant results on a 0.05 level.

### Subjective sleep quality

Participants had a median PSQI score of 4.0. Only 35% had a score above 5, indicating poor sleep quality. WI players had with 5.5 a median score slightly above the cut-off point. The ESS indicated with a median score of 7.0 no excessive sleepiness. Only 17% had a score above the cut-off point of 10. The D-MEQ revealed that the musicians belonged to the “intermediate” chronotype group. The FEPS questionnaire revealed that “focusing” and “brooding” were in a normal range for men and women, but male WI players displayed a critical unhealthy brooding score (Table 3).

**Table 3:** Subjective sleep data, presented are median (IQR) or numbers (%).

	ALL n = 29	WI n = 14	SI n = 15	*p*	<41 YEARS n = 14	≥41 YEARS n = 15	*p*
**PSQI (median)**	4.0 (3.0–7.0)	**5.5** (2.0–7.0)	4.0 (3.0–6.25)	0.980	4.0 (2.5–7.0)	5.0 (3.0–7.0)	0.390
**Score > 5**	10 (35%)	6 (43%)	4 (27%)	0.914	4 (29%)	6 (40%)	0.476
**ESS (median)**	7.0 (5.0–10.0)	8.0 (5.0–10.75)	7.0 (4.5–9.0)	0.527	9.0 (6.5–11.5)	6.0 (4.5–7.0)	**0.019**
**Score > 10**	5 (17%)	3 (21%)	2 (13%)	0.800	5 (36%)	0	n/a
**D_MEQ (median)**	48.0 (40.8–56.2)	48.5 (42.5–56.8)	47.5 (36.0–54.0)	0.403	42.0 (32.0–48.0)	53.0 (46.0–58.0)	**0.004**
**Scores 16–41**	7 (24%)	2 (14%)	5 (33%)	0.384	6 (43%)	1 (7%)	**0.032**
**Scores 42–58**	15 (52%)	8 (57%)	7 (47%)		6 (43%)	9 (60%)	
**Scores 59–86**	4 (14%)	2 (14%)	2 (13%)		1 (7%)	3 (20%)	
**FEPS-II (median)**							
**Women**	n = 8	n = 3	n = 5		n = 5	n = 3	
**Focusing**	15.0 (11.5–21.8)	18.0 (13.0–18.0)	13.0 (10.5–20.0)	0.250	13.0 (10.5–20.0)	18.0 (13.0–18.0)	0.250
**Brooding**	30.5 (27.3–34.5)	32.0 (28.0–32.0)	29.0 (21.5–34.0)	0.571	29.0 (21.5–34.0)	32.0 (28.0–32.0)	0.571
**Men**	n = 21	n = 11	n = 10		n = 9	n = 12	
**Focusing**	14.0 (11.5–19.0)	15.0 (13.3–19.0)	12.0 (10.5–19.0)	0.370	15.0 (10.3–19.0)	13.0 (11.5–19.0)	0.815
**Brooding**	26.0 (19.0–36.5)	**32.0 (24.8–37.3)**	20.0 (19.0–33.0)	0.167	**34.0 (30.3–39.5)**	20.0 (18.5–24.5)	**0.027**

WI, wind instrument; SI, string instrument; PSQI, Pittsburgh Sleep Quality Index, scores above 5 indicates bad sleep quality; ESS, Epworth Sleepiness Scale, scores above 10 indicate excessive daytime sleepiness and it is recommended to seek medical advice; D-MEQ, Morningness-Eveningness Questionnaire, German version, scores of 16–41 indicate evening chronotype, scores of 42–58 indicate intermediate chronotype, scores of 59–86 indicate morning chronotype; FEPS, "Fragebogen zur Erfassungallgemeiner und spezifischer Persönlichkeitsmerkmale Schlafgestörter", measuring trait aspects of sleep-related and general cognitive arousal and is divided into two subscales, separate for women and men: focusing and brooding. Focusing scores of 28–36 (women)/ 21–36 (men) and brooding scores of 38–49 (women)/ 31–45 (men) are considered unhealthy. *p*-values represent chi-square tests for dichotomous variables and Mann-Whitney U test for continuous variables, values above 0.05 are considered significant. Highlighted are scores in the unhealthy range and significant *p*-values.

Age had a significant effect on the ESS (*p = 0*.*019*), the D-MEQ (*p = 0*.*004*), and the brooding scale of FEPS-II for men (*p = 0*.*027*). The younger age group showed higher ESS scores than the older group which was strongest for WI players (<41 years, n = 5: 11.0, 9.5–12.0 vs. ≥41 years, n = 7: 5.0, 4.0–7.0; *p = 0*.*009*). The younger group consisted of significantly more “evening” chronotypes and the older group of more “morning” chronotypes. The brooding score among men within the young age group showed significant higher and unhealthy brooding scores than the older age group.

Comparing subjective sleep quality (PSQI) and objective SE from the PSG recordings, only SI players displayed a subjective sleep quality that mirrored the objective sleep quality: SI players with a high subjective sleep quality had a significant higher SE than SI players with a low subjective sleep quality (PSQI≤5, n = 10: SE = 86%, 81–91% vs. PSQI>5, n = 4: 70%, 62–81%; *p = 0*.*036*). WI players had high SE independent of their PSQI scores.

### Sleep-related respiratory variables and age

The participants (Table 4) had a median AHI of 2.1events/hour (IQR: 0.7–5.5, range: 0.2–18.4) and a median average oxygen saturation of 98% (IQR: 97–100, range: 95–100). Eight participants (4 WI, 4 SI) had at least a mild OSA (AHI≥5), none had a severe OSA (AHI≥30). There were no significant differences between instrument groups except for snoring with WI players snoring significantly more than SI players *(p = 0*.*01)*. Controlling for age, the Quade’s Test also showed no significant differences between instrument groups.

**Table 4:** Sleep-related respiratory variables, presented are median with interquartile range.

	ALL n = 29	WI n = 14 (48%)	SI n = 15 (52%)	*p*
**AHI (n/h)**	2.1 (0.7–5.5)	2.5 (0.6–5.7)	1.7 (0.7–5.5)	0.982
**HI (n/h)**	0.7 (0.2–3.8)	0.6 (0.1–4.1)	1.2 (0.4–3.5)	0.804
**AI (n/h)**	0.5 (0.3–1.9)	0.9 (0.2–2.0)	0.5 (0.3–1.8)	0.982
**Sp02-min (%)**	89.0 (86.0–92.5)	89.0 (86.0–91.5)	91.0 (85.0–93.0)	0.652
**Sp02-avg (%)**	97.9 (96.7–99.6)	97.7 (97.1–98.3)	99.3 (96.5–99.8)	0.146
**Arousal (n/h)**	15.2 (11.4–20.6)	13.9 (4.8–19.5)	15.4 (13.3–21.9)	0.146
**Snoring (% of TST)**	28.2 (4.7–38.4)	34.9 (23.3–44.8)	22.1 (0.0–32.8)	**0.01**

AHI, apnea-hypopnea index; n/h, number of events per hour; HI, hypopnea index; AI, apnea index; Sp02-min, minimum oxygen desaturation; Sp02-avg, mean oxygen desaturation; arousal, arousal index; TST, total sleep time; WI, wind instrument players; SI, string instrument players. *p*-values represent Mann-Whitney U test. Highlighted are significant results on a 0.05 level.

Including age in a more descriptive presentation by comparing the two age and instrument subgroups, we identified a different age pattern regarding sleep-related respiration for the instrument groups. All SI players with an AHI≥5 belonged to the older age group, while three of the four WI players with an AHI≥5 were of the younger age group. Within the older age group (≥41 years), SI players had a significant higher AHI than WI players (SI, n = 6: 5.6, 4.4–8.5 events/hour vs. WI, n = 9: 1.4, 0.4–3.1events/hour; *p = 0*.*01*). Contrary, within the younger group (<41 years), SI players had a significant lower AHI than WI players (SI, n = 9: 0.9, 0.4–1.2 events/hour vs. WI, n = 5: 5.5, 2.0–6.5events/hour; *p = 0*.*03*).

**Fig 1:**
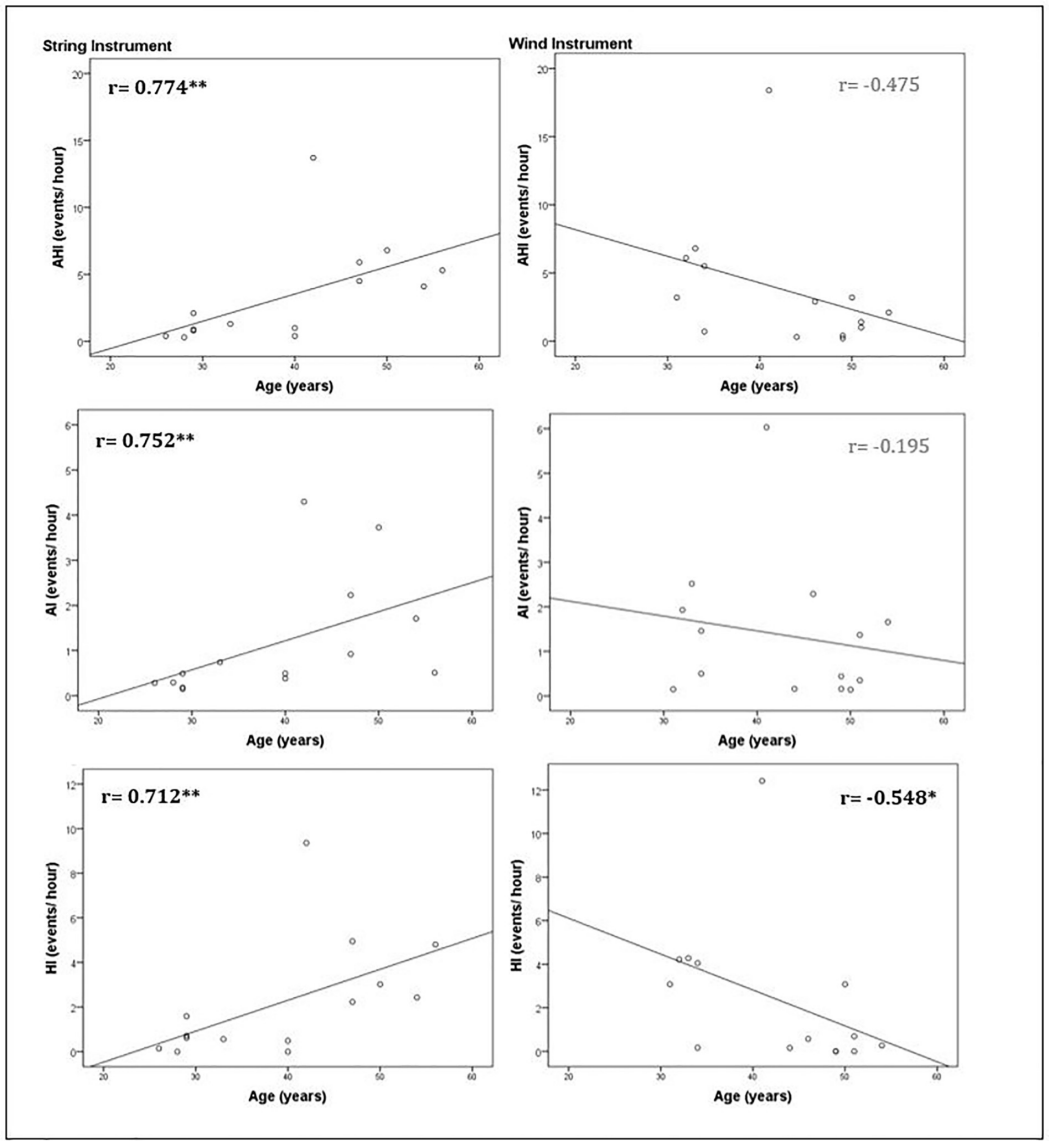
Correlations between age and breathing events separate for instrument groups. AHI, apnea-hypopnea index, AI, apnea index, HI, hypopnea index. Displayed are Spearman-Rho correlations, significant correlations are highlighted black and bold. Correlations are significant on a 0.05 level (*) and highly significant on a 0.01 level (**).

**Fig 2:**
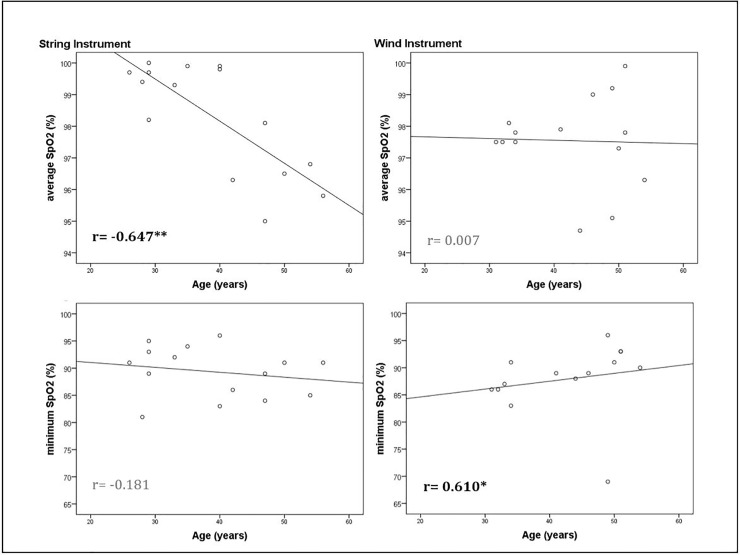
Correlations between age and oxygen saturation separate for instrument groups. Sp02, oxygen saturation. Displayed are Spearman-Rho correlations, significant correlations are highlighted black and bold. Correlations are significant on a 0.05 level (*) and highly significant on a 0.01 level (**).

The SI players showed highly significant positive correlations between age and sleep-related respiratory events (AHI: r = 0.774, *p = 0*.*001*; Hypopnea Index: r = 0.712, *p = 0*.*004*; Apnea Index: r = 0.752, *p = 0*.*002*) and a highly significant negative correlation between age and oxygen saturation (average oxygen saturation: r = -0.647; *p = 0*.*009*). The WI player showed opposite associations, negative correlations of age and sleep-related respiratory events (Hypopnea Index: r = -0.548; *p = 0*.*043*) and positive correlations between age and oxygen saturation (minimum oxygen saturation: r = 0.610; *p = 0*.*020*). While some parameters did not reach significance, they showed the same trend (Figs 1 and 2).

## Discussion

Our study is the first to record overnight polysomnographic sleep and sleep-related respiratory data with professional musicians. Against our original hypotheses, sleep showed only subtle signs of impairment and sleep-related respiration differences between instrument groups were modest. However, we did reveal a different age pattern of sleep-related respiration depending on instrument according to our original assumption. These findings provide first objective evidence to the assumption that habitual playing of a WI may have a protective effect on respiration. It reveals the need for more investigation as preventive training of the upper airway muscles may be of clinical relevance.

### Sleep in orchestra musicians

With a sleep efficiency above 85%, the musicians experienced a sleep quality in a normal range according to current literature [6,33]. Changes between instrument groups were small. However, WI players displayed a higher and more consistent objective sleep quality than SI players, even when controlling for age. Subjectively expressed lower sleep quality assessed by means of questionnaires did not manifest in WI players’ objective sleep quality assessment but in that of SI players’ objective sleep quality. WI players had an objectively seen good sleep quality no matter what they expressed subjectively. We were also able to collect important characteristics of the musicians including their sleep-related chronotype (D-MEQ) and personality traits that may affect sleep such as brooding and focusing (FEPS-II). Younger musicians presented a lower subjective sleep quality than older musicians. They consisted of more “evening” types and showed unhealthy brooding and daytime sleepiness. Reasons may include an increased need for sleep, family and economic reasons, but also the work situation. While prolonged stress levels of the profession may not have manifested in their sleep architecture, the young musicians may have felt it mentally. General and performance anxiety are commonly higher in music students than music professionals [34]. These results are important as they present individual differences in the group of musicians that may affect sleep. Future studies investigating sleep in musicians should include bigger sample sizes taking these differences into account.

The musicians presented a total sleep time of six hours. While there are individual differences regarding the amount of needed sleep [7], the recommended adult sleep amount is seven to nine hours to be fully awake and able to perform normally during daytime [8,35,36]. Also, the average recorded sleep duration is 7.3 hours in Singapore, 8.4 hours in the Netherlands, 7.8 hours in Germany, and 7.9 hours in the USA [37]. The reduced sleep duration of the musicians is comparable to that of shift workers and indicates sleep deprivation [38,39]. Already short-term sleep deprivation negatively affects sleepiness, mood, cognition, motor performance, and metabolic variables [7]. Studies even demonstrated an increased death rate by 1.7 times for men sleeping six hours or less compared to men sleeping seven or eight hours [40]. Compared to sleep data from current literature [33], the musicians showed a tendency of slightly more light sleep and less REM sleep (dream sleep) than healthy sleepers. Sleep deprivation usually causes a slow-wave sleep (deep sleep) rebound during the first undisturbed night and a REM rebound during the second undisturbed night [35,41]. Our results may suggest that the musicians needed additional nights to recover. However, changes were subtle and need further investigation.

### Sleep-related respiration and wind instruments

Sleep-related respiration including oxygen saturation and sleep-disordered breathing events was relatively good in the musicians. While statistically controlling for age, we did not identify differences between instrument groups. However, age was an influencing factor and comparing the two age and instrument subgroups in a more descriptive way, we found a strong interactive effect of instrument and age on respiration. Age is a risk factor for OSA and respiration tends to worsen with age [18]. While increased age indeed negatively affected sleep-related respiration in SI players, respiration for WI players improved with age. Breathing events decreased and oxygen saturation increased. As age and years of practice were positively correlated, we assume that improvement of sleep-related respiration is due to the habitual playing of the WI and therefore, the increased training of the upper airway muscles. This has been indicated by survey studies investigating the digeridoo and double reed WI [14–16]. Our sample of WI players included four double reed instruments (oboe, bassoon), but also five other woodwind instruments (flute, clarinet) and five brass wind instruments (horn, trumpet, trombone). However, a non-parametric Kruskal-Wallis Test showed no significant differences in sleep or respiration data between those groups. Due to the extremely small subgroup size we refrained from further analyses of the specific wind instrument types.

While playing a WI is not a feasible therapy concept, active training of the upper airway muscles is [22–24,42]. Therefore, these findings are of clinical relevance. Even though the respiratory pathology found was modest, we revealed an interesting age-related pattern on sleep-related respiration that was different for instrument groups. This needs to be explored in more detail within a larger group of musicians with a wider range of respiration events.

### Limitations

Our findings regarding sleep pattern and sleep-related respiration were modest. The study showed certain methodological limitations. These included especially the small sample size and the good sleep and respiration to begin with. We also recorded only one night and did not control for previous sleep or workload. However, full overnight polysomnography with orchestra musicians is unique and a particular strength of this study. In contrast to other studies [14–16], we assessed subjective and objective sleep and sleep-related respiration. Although findings are subtle and may not fully confirm our hypotheses, we were the first to objectively describe sleep in professional orchestra musicians and even revealed interesting signs of changes in sleep parameter and sleep-related respiration in relation to instrument and age. This study clearly identifies the need for further investigation and presents the motivation and foundation to build upon. Future research should include a larger participation, possibly including differentiation of WI and upper airway muscles workload.

## Conclusion

Our results revealed subtle but interesting changes in sleep parameter and sleep-related respiration in orchestra musicians, mediated by instrument and age. WI players seemed to have a better sleep quality and respiration than SI player. We identified an opposing age impact on sleep-related respiration between instrument groups. While respiration in SI players decreased over time, it improved in WI players. These findings may be of clinical relevance and strengthen the assumption that playing a WI and training the upper airway muscles may protect respiration.

## Supporting information

S1 File Masterspreadsheet with raw data information. (PDF)
