# Supplementary material for: Overnight polysomnography and the recording of sleep and sleep-related respiration in orchestra musicians – possible protective effects of wind instruments on respiration
Source: PLoS One. 2020 Apr 15;15(4):e0231549. doi: 10.1371/journal.pone.0231549 (PMC7159236; doi:10.1371/journal.pone.0231549)
Supplement: S1 File — (PDF) [file pone.0231549.s001.pdf]

Pb	BMI (kg/m^2)	gender m=male f=female	age (range of years)	instrument 1=string 2=wind	instrument (text)	Instrument (text details)	allergies 1=yes 0=no	allergies (text)	alcohol 1=yes 0=no	alcohol (text)	medication 1=yes 0=no
1	26	w	28-56	1	string	violin	1	hayfever		0	0
2		m	28-56	2	wind	bassoon	0	0		0.2 l (10pm)	1
3	27	m	28-56	2	wind	clarinet	0	0		0.05 ml anis spirit	0
4		m	28-56	1	string	viola	1	hayfever, cat hair, pollen		0	0
5	25	m	28-56	1	string	cello	1	cat hair		0.5 l beer (10pm)	0
6		m	28-56	1	string	cello	0	0		0	0
7	19	m	28-56	1	string	violin	1	hayfever		0	1
8	23	m	28-56	1	string	viola	1	penicillin	1	0.2 l vine (8pm)	0
9	27	f	28-56	2	wind	flute	0	0			1
10	21	m	28-56	1	string	cello	1	penicillin, tropical fruits, hayfever	1	1 l beer (till 1am)	0
11	25	m	28-56	1	string	violin	1	hayfever, cat hair, mold, birch	0	0	0
12	20	f	28-56	1	string	bass	0	0	0	0	1
13	21	m	28-56	1	string	violin			1	0.2 l vine (10pm)	0
14	26	f	28-56	1	string	viola	1	ciprobay	0	0	0
15	22	m	28-56	2	wind	oboe	0	0	0	0	0
16	30	m	28-56	1	string	violin			1	0.2 l vine	1
17	27	m	28-56	1	string	bass	1	hayfever	1	0.2 l prosecco	1
18		f	28-56	1	string	violin	0	0	0	0	0
20	24	f	28-56	1	string	violin	0	0	0	0	1
21	22	f	28-56	2	wind	flute	0	0	0	0	0
22	25	m	28-56	2	wind	trumpet	0	0	0	0	0
23	25	m	28-56	2	wind	horn	0	0	0	0	0
24	24	m	28-56	2	wind	oboe	0	0	0	0	1
28	29	m	28-56	2	wind	clarinet	0	0	0	0	0
30	25	m	28-56	2	wind	trombone	1	grass, pollen	0	0	0
31	23	m	28-56	2	wind	flute	1	cat hair	1	0.2 l vine	0
32	34	m	28-56	2	wind	horn	1	grass, pollen, penicillin	0	0	1
33	20	f	28-56	2	wind	oboe	0	0	1	1 beer	0
34	22	m	28-56	2	wind	horn	1	grass, pollen	0	0	0

medication (text)	surgery 1=yes 0=no	surgery (text)	sport 1=yes 0=no	Playing Instrument (days/ week)	Playing Instrument (years)	light off (time)	light on (time)	SE (%)	TIB (min)
0	0	0	1	4	19	00:58:00	08:05:00	88	427
20 mg nexium	0	0	1	4	33	00:10:00	06:10:00	89	360
0	0	0	0			01:00:00	08:30:00	85	450
0	1	nasal septum, tonsillectomy, Cholezystektomie	0			03:00:00		91	
0	0	0	1		41	23:45:00	06:21:00	92	396
0	0	0	0			01:30:00		79	
Delix	0	0	0					89	
0	1	tonsillectomy	0	3	48	00:45:00	08:00:00	86	435
L-thyroxin (eye pressure)	1	eye surgery, thyroid	0			00:25:00	08:00:00	86	455
0	0	o	0		27	02:15:00	08:15:00	66	360
0	1	variceal surgery	1		37	23:40:00	06:30:00	82	410
antibaby pill	0	0	1	5	15	00:54:00	08:15:00	95	501
0	0	0	0		23	01:30:00	11:00:00	84	570
0	0	0	1		20	01:04:00	08:00:00	87	416
0	0	0	0	2.5	21	23:55:00	07:00:00	92	425
homeopathic	1	cervical spine, laryngeal surgery, ENT, meniscus	1	1	45	00:15:00	08:55:00	65	520
ezetrol	0	0	0		27	23:35:00	07:00:00	7	445
0	0	0	0			01:30:00		74	
nasal spray	1	tonsillectomy	0			00:33:00	07:15:00	85	402
0	1	ENT	0			00:30:00	07:15:00	89	405
0	1	inguinal hernia	1	2.5	25	23:45:00	08:30:00	89	525
0	1	variceal surgery	0	1	23	23:50:00	06:20:00	78	390
salbutamol 1x/d	1	nasal septum deviation	1	2	25	00:30:00		89	
0	0	0	1	2.5	32	23:30:00	07:08:00	94	458
0	0	0	1	2.5	21	00:30:00	07:00:00	93	390
0	1	nasal septum, dental extraction	1	1	38	00:15:00	08:00:00	92	465
syntaris (flumisolid) 1-0-0 (nasal spray)	1	tonsillectomy, dental extraction	0	2.5	23	01:40:00	08:10:00	95	390
0	0	0	1	2	27	01:29:00	08:13:00	96	404
0	1	tonsillectomy	1	2	31	00:20:00	08:50:00	82	510

TST (hours)	TST (min)	SL2 (min)	REM_L (min)	WASO (min)	NREM1 (% of SPT)	NREM2 (% of SPT)	SWS (% of SPT)	REM (% of SPT)	AHI (events/h)	HI (events/h)	AI (events/h)	SpO2_min (%)	SpO2_avg (%)	arousal (number/h)
06:53	413	47	92	9	9	49	26	15	0	0	0	81	99	18
05:47	347	27	68	8	3	45	38	12	1	1	0	93	98	22
07:23	443	28	74	53	17	51	10	12	0	0	0	69	95	20
06:17	377	13	65	27	2	62	9	21	5	2	2	89	98	22
05:27	327	7	6	21	7	52	18	19	6	5	1	84	95	11
05:17	317	27	87	29	5	41	38	8	0	0	0	96	100	11
08:02	482	13	116	47	10	41	26	15	1	0	0	83	100	24
07:03	423	65	62	11	8	50	24	18	4	2	2	85	97	17
06:54	414	56	71	20	10	50	21	17	1	0	1	93	100	11
05:24	324	99	86	16	12	60	14	12	1	1	1	92	99	12
05:50	350	13	67	55	5	45	22	16	5	5	1	91	96	14
07:40	460	12	52	14	4	42	42	9	0	0	0	91	100	21
08:12	492	66	74	46	15	36	26	18	2	2	0	89	98	14
06:34	394	25	125	36	12	47	20	13	1	1	0	93	100	15
06:30	390	8	59	23	16	37	26	16	3	3	0	86	98	5
05:37	337	6	45	95	14	38	3	22	7	3	4	91	97	26
06:31	391	8	80	41	11	33	35	12	14	9	4	86	96	15
05:39	339	80	74	43	7	49	16	19	1	1	0	95	100	13
06:10	370	34	121	32	5	52	16	20				94	100	28
06:10	370	14	60	35	5	45	23	19	0	0	0	96	99	23
07:44	464	49	71	4	7	44	27	22	7	4	3	87	98	15
05:28	328	34	155	27	11	55	19	7	18	12	6	89	98	16
05:41	341	33	138	6	7	51	29	12	6	4	2	86	98	19
07:09	429	7	71	16	6	63	13	14	3	3	0	91	97	18
06:03	363	7	103	22	3	56	11	26	0,7	0	1	91	98	8
05:29	329	5	61	28	7	61	12	13	2	0	2	90	96	13
06:11	371	1	69	15	3	61	14	19	6	4	1	83	98	2
06:27	387	2	62	13	9	65	4	20	0	0	0	88	95	3
06:59	419	28	146	70	16	48	3	19	3	1	2	89	99	4

snoring (% of TST)	SPT (min)	PSQI	ESS	FEPS-focusing	FEPS-brooding	D-MEQ
0	418	4	9	11	35	41
49	454		6			
39	490	2		13	18	42
16	402	4	1	12	19	59
0	345	3	7	11	19	48
66	344	4	3	9	19	42
27	527					
23	425	3	9	12	26	53
0	427	5	5	13	49	44
0	330	7	8	20	40	38
9	402	5	7	14	15	57
0	472	4	6	13	27	47
29	521	10	3	23	45	26
38	426	3	11	17	33	48
41	410	3	10	9	30	48
36	436	3	5	10	20	49
33	420	6	7	18	21	68
0	375	8	7	10	29	30
22	401	4	14	23	16	29
44	402	7	3	18	28	40
25	466	2	12	15	31	59
52	354					
0	347	7	12	14	35	49
18	444	6	5	24	23	62
48	383	1	9	16	38	34
31	355	7	4			57
35	385	2	11	15	33	44
28	399	8	10	24	32	53
35	482	11	7	20	51	56
